# Comparison of Microleakage in Nanocomposite and Amalgam as a Crown Foundation Material Luted with Different Luting Cements under CAD-CAM Milled Metal Crowns: An In Vitro Microscopic Study

**DOI:** 10.3390/polym14132609

**Published:** 2022-06-27

**Authors:** Amina, Geeta Rajput, Saad Ahmed, Saurabh Chaturvedi, Mohamed Khaled Addas, Tushar Vitthalrao Bhagat, Vishwanath Gurumurthy, Saeed M. Alqahtani, Mohammed A. Alobaid, Ebrahim Fihaid Alsubaiy, Kanishk Gupta

**Affiliations:** 1 Department of Prosthodontics, Crown & Bridge Dr Ziauddin Ahmad Dental College, Hospital Aligarh Muslim University, Aligarh 202002, India; amina6468.aa@gmail.com (A.); geetarajput70@gmail.com (G.R.); 2 Department of Oral Pathology, Microbiology Rama Dental College, Hospital and Research Center, Kanpur 208024, India; saad.ahmad84@gmail.com; 3 Department of Prosthodontics, College of Dentistry, King Khalid University, Abha 62529, Saudi Arabia; 4 Department of Prosthetic Dentistry, College of Dentistry, King Khalid University, Abha 62529, Saudi Arabia; maddas@kku.edu.sa (M.K.A.); or smaalqahtani@kku.edu.sa (S.M.A.); or Alsbuay@kku.edu.sa (E.F.A.); 5 College of Dentistry, Prince Sattam Bin Abdulaziz University, AlKharj 16245, Saudi Arabia; 7508tvb@gmail.com; 6 Department of Dental Technology, College of Applied Medical Sciences, King Khalid University, Abha 61421, Saudi Arabia; vishwa.stem@gmail.com; 7 Restorative Dental Science Department, College of Dentistry, King Khalid University, Abha 62529, Saudi Arabia; alobaid85@outlook.com; 8 Department of Dental Education, College of Dentistry, King Khalid University, Abha 62529, Saudi Arabia; 9 Department of Periodontology, Dentistry Program, Batterjee Medical College, Jeddah 21442, Saudi Arabia; drkanishkgupta@gmail.com

**Keywords:** microleakage, crown foundation materials, luting cements, CAD-CAM, milled crown, adhesive cement

## Abstract

Microleakage is a persistent problem despite advancement in materials and techniques in fixed prosthodontics. This leads to the importance of sound crown foundation material and luting agents used to maintain the marginal seal. The literature is deficient with studies, comparing microleakage under various crown foundation materials and luting agents, especially with CAD-CAM (computer-aided design and computer-aided manufacturing) metal crowns. This study was aimed to compare microleakage in a nanocomposite/dentinal bonding agent and amalgam/cavity varnish as crown foundation materials luted with two different luting cements: resin-reinforced glass ionomer cement and self-adhesive resin cement, under both dry and contaminated conditions. A hundred intact, caries-free human molars were prepared to receive crown foundation material and extra coronal restorations. Amalgams with cavity varnish and nanocomposites with dentinal bonding agent in both ideal and contaminated conditions were used as crown foundation materials. After restoration, each sample was cemented with a CAD-CAM milled metal crown using two different luting agents—resin-reinforced glass ionomer cement and self-adhesive resin cements both in ideal and contaminated conditions. Cementation was followed by thermocycling of samples, immersion in erythrosine B dye, embedding in clear auto polymerizing acrylic resin and sectioning to evaluate microleakage using stereomicroscope. The mean microleakage between different luting cements on the experimental side of the facial surface was 137.64 μm and 211.01 μm for resin-reinforced GIC and for self-adhesive resin cement was 119.78 μm and 150.42 μm, under ideal and contaminated condition, respectively. There was a significant difference in mean micro-leakage between different crown foundation material and cement groups used in the study. The composites and amalgam, both when used as crown foundation material and luted with use of technically advanced CAD-CAM metal crown with self-adhesive resin cement (in both ideal or contaminated condition), showed less microleakage than in resin-reinforced glass ionomer cement. Overall, the self-adhesive resin cement showed comparatively reduced microleakage in all combinations with different crown foundations. Thus, this combination can be used in daily clinical practice to provide better protection from further decay.

## 1. Introduction

Microleakage is the leaching of fluids in the oral cavity, comprising debris and bacteria between the tooth and the restorative material or tooth and cement layer [1]. Microleakage occurring along the interfaces has been related to secondary caries, hypersensitivity [2] and pulpal problems [3]. Microleakage can be related to cement disintegration through dissolution in oral fluids or shrinkage on setting [4]. The first step in the rehabilitation of a grossly destroyed tooth is placement of an ideal crown foundation material. Different materials that can be used as crown foundation materials are amalgam, composite, cast gold, and glass ionomer cement. Amalgam is considered the primary restorative material of choice as a crown foundation because of its cost effectiveness, strength, and ability to be restored in a wet field. Composite is another choice of material, as it can be polymerized easily and rapidly, which is helpful for the clinician for building the tooth, prepare it to receive casting and make an impression in one appointment [5]. Its added advantages also includes its ability to bond to tooth structure, thermally nonconductive nature, close colour matching with the tooth, no mercury, and biocompatibility [4,5]. Nanocomposites has been a major breakthrough in the last few years as filler particles fall in the range of 0.1–100 nanometers (nm) [6]. Due to wide-sized distribution and reduced filler particle size, the higher amount of filler load can be achieved, thus reducing shrinkage due to polymerization and hence microleakage [7].

In many instances, extra coronal restorations do not seat completely despite being precisely fabricated by the laboratory. To fill this marginal void and hence inhibit microleakage at the crown–tooth interface, luting cements are used. According to Tjan and Chiu [8], the microleakage in a crown is more affected by the type of luting agent than the core build-up material.

An ideal luting agent should possess favourable compressive and tensile strength, it should provide a stable bond between two different metals, have good fracture strength so as to avoid the dislodgement due to failure of adhesive or cohesive force/bond, exhibit appropriate viscosity and adequate film thickness, should be able to wet restoration and tooth, have sufficient working time, should not get disintegrated in the mouth and have tissue compatibility. It is important for the cement to possess a high modulus of elasticity in order to prevent microleakage [9]. The value of the modulus of elasticity of these luting agents should lie in between indirect restorative material and dentin [10]. Cementing agents commonly used in in vitro studies are zinc polycarboxylate cement, zinc phosphate cement, glass ionomer cement, resin-reinforced glass ionomer cement and adhesive resin cements. Glass ionomer cement is an adhesive cement that provides greater resistance to microleakage than non-adhesive cements. However, the problem in this cement is its high solubility and susceptibility to moisture if exposed to moisture during the initial setting period. To overcome the problems of solubility and lack of adhesion, newer resin cements have been introduced [11,12]. However, a high coefficient of thermal expansion and hence polymerization shrinkage are major concerns with these resins containing luting agents. Stresses developed due to polymerization shrinkage are counteracted by hygroscopic expansion. However, the amount of compensatory hygroscopic expansion which would be sufficient to navigate the effect requires hours or days to take place while polymerization shrinkage occurs immediately. Therefore, to reduce the effect of the collective forces of polymerization shrinkage, immediate bonding to dentin is crucial. Resin-reinforced glass ionomer cement is commonly used nowadays as these cements along with containing beneficial properties of glass ionomer cement such as fluoride release, has improved physical and chemical properties similar to adhesive resin cement [13]. Incorporation of polymerizable functional groups impart more rapid curing in comparison to conventional GIC, decrease moisture sensitivity and increase early strength of cement [6]. The behaviour or performance of different cements and restorative materials to wet, or contaminated environment is a operatory demand that needs to be addressed with through investigation. Some cements like zinc phosphate and glass ionomer cement are shown to be more susceptible to degradation by presence of moisture as compared to resin cements and resin containing cements. Various agents employed for evaluating microleakage include dyes, air pressure, bacteria and radioactive isotopes [14]. Out of these methods, the dye method is chosen for this study because of its ease of use and simplicity of the procedure. Another advantage of this technique is that it demonstrates microleakage in contrasting colour to both tooth and restoration without any further use of chemicals or tests.

Dental restorations regularly face extreme changes in the temperature of the oral environment. For simulating such situations of the oral cavity, the restorations are subjected to artificial aging by thermocycling loading. CAD-CAM crown fabrication assures an instantaneous feedback related to the quality of the preparation through the digitalised procedure of scanning and digital designing by software program [15]. However, assessment of microleakage under such crown can provide important critical information about the future success of the crown, as microleakage may result in secondary caries and premature failure [15,16]. After aggregating these points together, it was found that there is a lack in literature describing the comparison of microleakage in various foundation materials and luting agents. In the present study, for the first time we compared the commonly used crown foundation materials and luting agents, especially with CAD-CAM metal crown, as the use of digital dentistry is increasing day by day and it should be assessed thoroughly to obtain a better understanding. Thus, this study was planned with the aim to compare microleakage in nanocomposite/dentinal bonding agent and amalgam/cavity varnish as crown foundation materials luted with two different luting cements: resin-reinforced glass ionomer cement and self-adhesive resin cement under both dry and contaminated conditions. The null hypothesis formulated was that there would be no difference in microleakage between the compared variables in the study.

## 2. Materials and Methods

**Figure 1:**
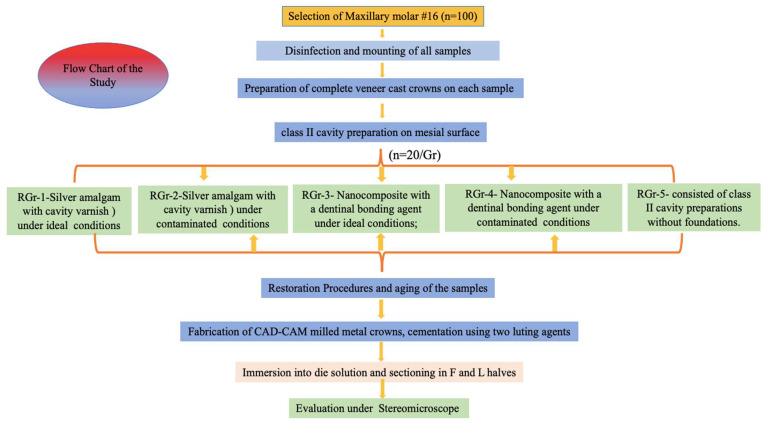
Flow chart of study.

The present cross-sectional study was conducted in the Department of Prosthodontics, College of Dentistry. To standardize the study protocol, a synchronized flowchart was prepared for fabrication of various samples (Figure 1). Ethical clearance was obtained from the institute’s ethical committee.

### 2.1. Selection and Preparation of Samples

One hundred human maxillary molars were collected from the institute teeth bank and were cleaned, disinfected, and stored in 0.1% thymol solution during the experiment at all the times except during preparation and testing. The teeth were screened to rule out any cracks, craze lines, defects, immature root apices, craze lines and fractures. The selected teeth were mounted on the mounted in dental plaster (Dentico, Neelkanth, Pvt, Ltd., Boranada, India). Tooth preparations for complete veneer cast crowns were performed on each tooth. The chamfer finish line was prepared with total occlusal reduction of 1.5mm and angle of convergence was kept at 6 degrees. On the mesial surface, a class II cavity was prepared with 4 mm width in bucco-lingual dimension and depth 2 mm of each tooth. Five restorative groups (RGr) (n = 20/group) were formed based on the two restorative crown foundation materials and restorative conditions. RGr-1-Silver amalgam (fine grain DPI, Bombay Burmah Trading Corporation, Ltd., Mumbai, India) with cavity varnish (Copal F, Prevest DenPro, Jammu, India) under ideal (dry, clean working field); conditions; RGr-2 Silver amalgam (fine grain DPI, Bombay Burmah Trading Corporation, Ltd., Mumbai, India) with cavity varnish (Copal F, Prevest DenPro, Jammu, India) under contaminated conditions; RGr-3-Nanocomposite (Tetric N-Ceram, Ivoclar Vivadent, Schaan, Liechtenstein) with a dentinal bonding agent (Tetric N-Bond, Ivoclar Vivadent, Schaan, Liechtenstein) under ideal (dry, clean working field) conditions; RGr-4- Nanocomposite (Tetric N-Ceram, Ivoclar Vivadent, Schaan, Liechtenstein) with a dentinal bonding agent (Tetric N-Bond, Ivoclar Vivadent, Schaan, Liechtenstein) under contaminated conditions. Gr-5-consisted of class II cavity preparations without foundations. Similarly same samples were segregated in 4 cementing groups (CGr) (n = 25/group) based on the type of luting agent used and type cementing conditions-CGr-1 Resin reinforced Glass Ionomer Cement Glass Ionomer Cement (Fuji CEM, GC, Tokyo, Japan) under ideal condition, CGr-2 Resin reinforced Glass Ionomer Cement under contaminated condition; CGr-3 Self-adhesive Resin cement (Bifix SE, Voco, Rosemont Drive, Suite, Indian Land, USA) under ideal condition; CGr-4 Self-adhesive Resin cement under contaminated condition.

### 2.2. Restoration Procedures and Aging of the Samples

Each tooth was restored following the pre-decided procedure finalized by the chief researcher. To restore teeth with amalgam/cavity varnish, - in RGr- 1–2 layers of cavity varnish were applied and lightly air dried before amalgam placement. Amalgam was properly triturated; excess mercury was squeezed out using chamois leather, filled in the prepared cavity and condensed well against the walls after placement of matrix band and retainer. Burnishing of restorations was performed using ball burnisher. In RGr-2, the same steps were followed for the contaminated group, except a thin layer of artificial saliva was applied after varnish application to cavity walls. In RGr-3 and 4, where the composite/dentinal bonding agent is to be used, a cleaned tooth was used in RGr-3 and saliva contaminated samples were used in RGr-4. In both groups, all-Etch technique was used and solution was applied for 15 s, then samples were thoroughly rinsed and dried. Five layers, one after the other, of primer were applied and dried. Then, dentin/enamel bonding resin was applied in thin layer and light cured, and following this, the composite was placed in increments and light cured. The vertical layering technique was used for composite restoration as it was proved to be beneficial in microleakage as compared to the oblique technique. In the contaminated group, same steps were followed, except that saliva was placed on the tooth and lightly air thinned before and after the application of the dentinal bond agent. After restoration of samples, the refinement of tooth preparation was done so as to place the finish line 1.5 mm gingival to each restoration and on enamel. Initially, aging of the samples with restoration was carried out by placing samples inside water and thymol solution for 2 weeks without provisional restorations, during this time the CAD-CAM metal crowns were fabricated using milling technique, final aging of samples were done using thermocycling unit, after cementation. These time samples with cemented metal crown were immersed in water baths at 5 °C and 55 °C with 12 s dwell time for 150 cycles.

### 2.3. Fabrication of CAD-CAM Milled Metal Crowns, Cementation, and Sectioning

**Figure 2:**
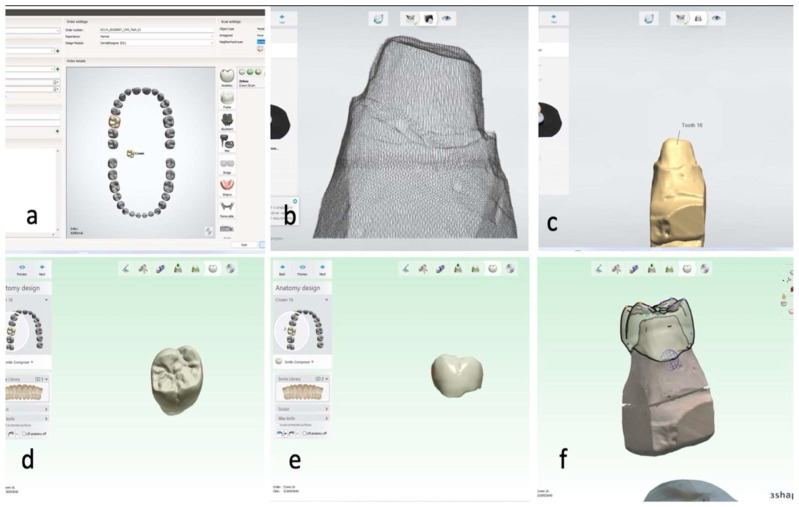
Representative images showing CAD process of metal crown fabrication. (**a**) Selection of tooth to be fabricated in 3-shape CAD software; (**b**) initial scanning process of mesh formation of sample by desktop scanner (Ceramill Map 400; AmannGirrbach); (**c**) final scanned image of the sample in 3-shape CAD software; (**d**) occlusal view of the designed crown; (**e**) buccal view of the designed crown; and (**f**) designed crown over the die, finalized by the 3-shape CAD software.

The prepared models were scanned using a desktop scanner (Ceramill Map 400; AmannGirrbach, Herrschaftswiesen, Koblach, Austria) and the data was transferred in standard tessellation language (STL) format to a CAD software (3Shape A/S, Holmens Kanal 7, Copenhagen, Denmark), for the design and the fabrication of full coverage metal crown. The virtual crowns with 0.8 μm luting space were designed for each sample. The metal crowns were fabricated using a 5-axis milling machine (Ceramill Matik, Amann Girrbach, Herrschaftswiesen, Koblach, Austria) and metal blocks (CoCr blanks Amann Girrbach), following manufacturer’s recommendation (Figure 2).

The artificial crowns were then cemented in 4 groups (CGr) as described previously. For simulating the contamination, the saliva was applied on the tooth as described previously. Finger pressure was used to hold the crown in its position during cementation of metal crowns. The teeth then were stored in room-temperature water for 2 weeks before thermocycling.

**Figure 3:**
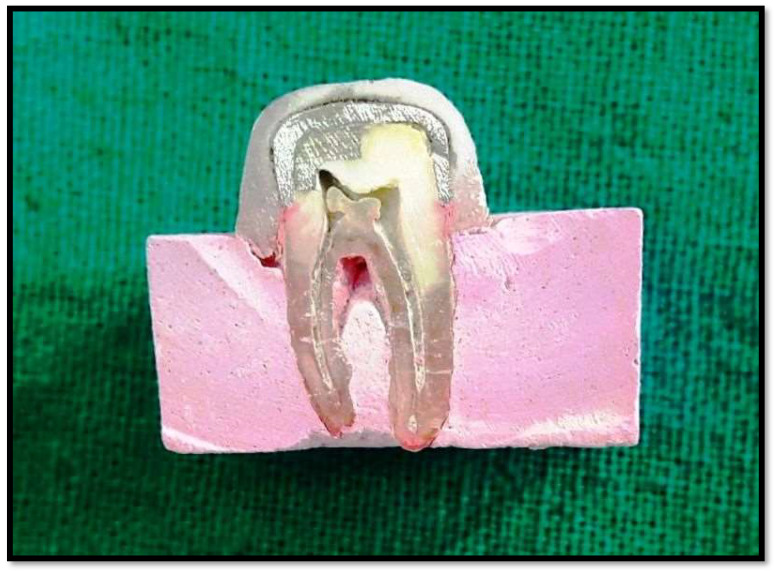
Tooth sectioned mesiodistally using precision cutting machine.

All the samples were aged using thermocycling for 150 cycles of 12 s dwell time at 5 °C and 55 °C. After this the samples with cemented metal crowns were immersed in erythrosine B solution for 24 h and rinsed properly. Following this the coronal surfaces of the crowns were embedded in clear acrylic resin. Then, with the help of precision cutting machine (BUEHLER Worldwide, Waukegan Road, Lake Bluff, IL, USA) each sample was cut in mesiodistal direction as shown in Figure 3.

**Figure 4:**
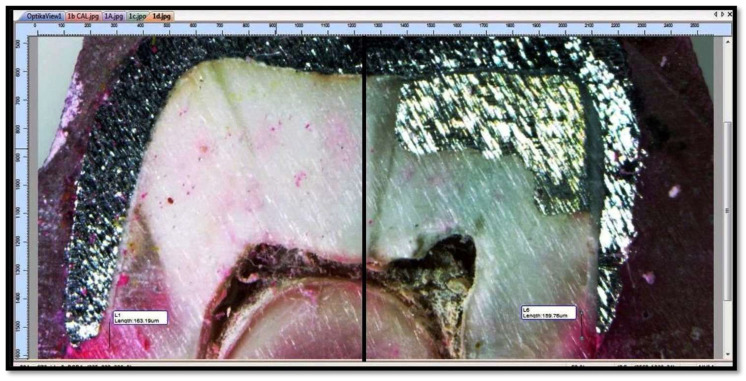
The tooth half with restoration was the experimental side; the other half served as the control (Each sample was studied by diving into facial part marked as F and the lingual part as L. Each F and L part were divided into a right and left half for the digitizing process. The sample half (divided by a line at the centre) with restoration (amalgam or composite) was considered as experimental half and other half was marked as control.

The surface of the tooth having the facial part was marked as F and the lingual part as L. Each F and L part was divided into a right and left half for the digitizing process. The sample half having the restoration was considered as the experimental half, and the remaining half was marked as control (Figure 4).

**Figure 5:**
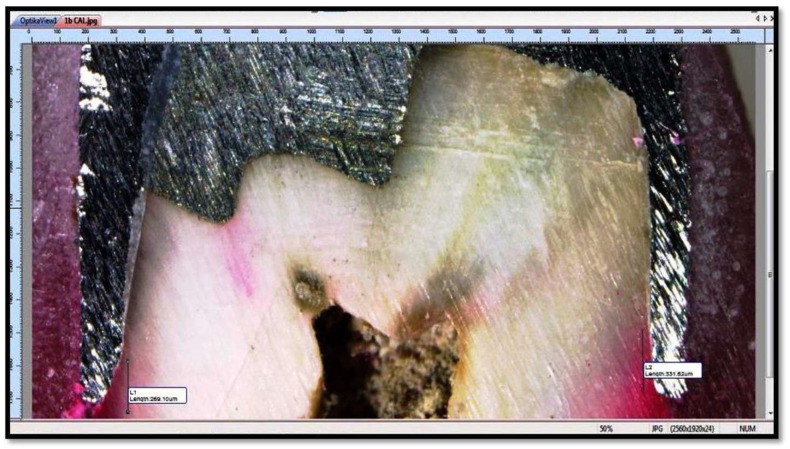
Microleakage was observed at tooth-metal crown and tooth-foundation material interfaces (Each sample was studied under microscope with 40X magnification to check for microleakage between tooth-metal crown and tooth-foundation material (amalgam or composite) interfaces.).

Microleakage was then observed at tooth coping and tooth foundation material interfaces at 40X using Stereomicroscope (OPTIKA SZM-LED 2) with attached camera (OPTIKAM B5) and digitized using software View Version 7.3.1.7 (OPTIKA, Via Rigla, Ponteranica (BG)—Italy) after capturing images of the samples (Figure 5).

## 3. Results

The microleakage of each F and L half on all teeth was evaluated with Wilcoxon sign-rank test. It was concluded that there was no significant difference in mean micro-leakage between facial and lingual surface under crown foundation materials (*p* value = 0.068) and luting cements (*p* value = 0.953). Microleakage between different groups of luting cements and crown foundation materials was evaluated with Kruskal–Wallis test.

**Figure 6:**
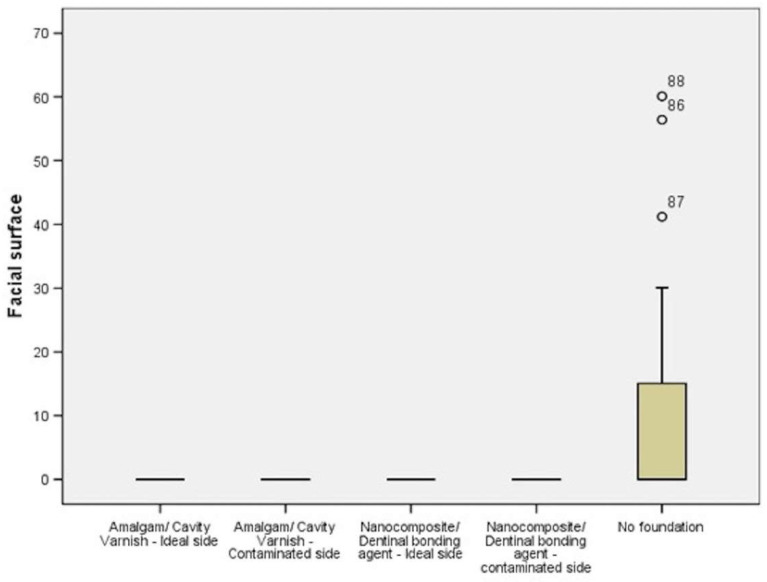
Box plot showing comparison of microleakage between different crown foundation materials on facial surface.

On evaluation of microleakage in the foundation material it was found that there was no microleakage with RGr-1 Amalgam/Cavity Varnish—Ideal group, RGr-2-Amalgam/Cavity Varnish—Contaminated group, RGr-3-Nanocomposite/Dentinal bonding agent—Ideal group, RGr-4-Nanocomposite/Dentinal bonding agent—contaminated group and microleakage was found to occur in RGr-5 without crown foundation material on both buccal and lingual side (*p* value < 0.001) (Table 1) (Figure 6).

**Figure 7:**
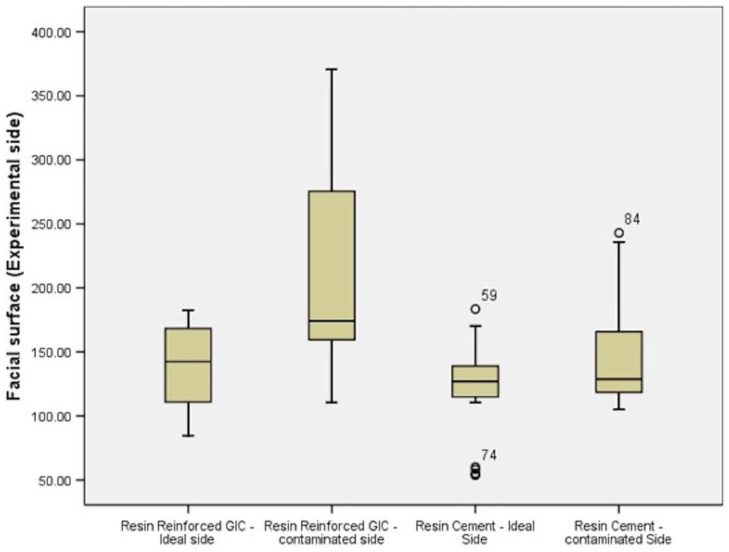
Box plot showing comparison of microleakage between different luting cements on experimental side of facial surface.

For both sides, i.e., control side and experimental side, evaluation was done individually. Both sides showed that the highest microleakage was in resin-reinforced glass ionomer cement under contaminated conditions and least was in self-adhesive resin cement under ideal conditions. The same order was followed in groups ranked from least to greatest microleakage (Table 2) (Figure 7).

## 4. Discussion

The ultimate success of an extra coronal restoration depends on the precision with which it is fabricated, physical and biomechanical properties of luting agent and presence of sound foundation material. Microleakage can be explained as movement of microbes, fluids, ions and bacteria between the cavity surface and restoration, which cannot be identified clinically [15]. It results in an unesthetic look in the form of staining around the margins, postoperative sensitivity, secondary caries, restoration failure, pulpal damage, and partial or total loss of restoration [16,17].

The first and most important step determining the success of restoration is the placement of sound foundation materials. It is critical to select a crown foundation material of appropriate strength if the tooth structure is grossly destroyed. On this basis, both amalgam and composites may be indicated. Nanocomposites are proved to exhibit less polymerization shrinkage in comparison to conventional composites, hence exhibit less microleakage [7]. No study has yet been conducted to describe direct comparison of their ability to prevent microleakage with another important crown foundation material, amalgam, which has self-sealing capacity to prevent microleakage. The present study was novel in itself, as for the first time, any study compared the said combinations with luted CAD-CAM milled crowns. Tjan et al. [8] in their study concluded that amalgam is better than composite and glass ionomer cement as crown foundation material in terms of prevention of microleakage, if the restoration margin cannot extend more than 1 mm from the foundation–tooth junction. Ease of manipulation is another important factor in selection of restoration material.

With developments in the field of dentistry and material science, a wide variety of restorative materials are available, and the nano-ionomers and nanocomposites are the most recent in the list. Nanotechnology has become very popular and engaged in our daily practice of life and dentistry. It can produce the materials and structures in the range of 0.1 to 100 nm by various physical and chemical methods. By adding the nanofiller, the composite can be made with aesthetics, strong enough to withstand forces easy, and have improved polishability and enhancement characteristics [18].

Therefore, in the present study, comparison of microleakage was made between amalgam, when used with cavity varnish and nanocomposites, and when used with dentinal bonding agents. It was found in previous studies that these combinations are effective in preventing microleakage. The results of the study showed that the amalgam and cavity varnish and composite and dentine bonding agent combination had no microleakage, while 12.52 μm mean microleakage was noticed in samples with no crown foundation material, which was in association with the studies of Barber et al. [19] and Andrews et al. [20]. Barber et al. [19] in their study showed that copal resin varnish is effective in sealing amalgam margins against penetration of ionic and molecular tracers. Andrews et al. [20] also demonstrated a reduction in microleakage in amalgam restorations filled after application of cavity varnish, irrespective of the alloy used. The resin-reinforced GIC group had more microleakage than self-adhesive resin cement group, both in ideal and contaminated conditions. This result of our study was in agreement with the study of Vargas et al. [11] and Ebadian et al. [21]. Ebadian et al. studied the microleakage of restorations cemented with RelyX™ Ultimate, RelyX™ Unicem, GC Gold Label, and Hoffmann cements, finding that Hoffmann zinc phosphate cement obtained higher microleakage values (5.00 ± 2.000 mm) followed by GC Gold Label glass ionomer cement (2.71 ± 1.976 mm) and finally by the resinous cements RelyX™ Unicem (2.14 ± 1.952 mm) and RelyX™ Ultimate (0.86 ± 1.215 mm). RelyX™ Ultimate is an self-adhesive resin cement and is used in combination with an adhesive (i.e., Scotchbond™ Universal Adhesive) to achieve adequate bond strengths [21].

For restoration with nanocomposites, a vertical layering technique was used in the present study, as it was proved by Bagis et al. [22] that the nanohybrid composites showed better results with the vertical layering technique compared to oblique layering for enamel margin in prevention of microleakage. Even though various contradictory results are enumerated in the literature regarding the technique of nanocomposite restoration, where few authors had mentioned that there was no influence of different composite placement techniques on microleakage, while Eakle and Ito [23] found that the diagonal insertion technique had the most leak-free margins when the proximal box ended on enamel. To avoid the bias and to standardise the procedure, the vertical layering technique was used as it was easy to use and the operator was comfortable with it, since the aim was to assess the effect of foundation material, uniformly single technique was used for restoration.

Besides the type of composite used, the success and longevity of restoration are determined by the micromechanical bond between the tooth surface and composite resins, which is reinforced by the dentine bonding agents. The 6th generation bonding systems achieve a strong bond to enamel and dentin using only “one solution.” These materials are one-step bonding system. Ansari [24] evaluated the strength of composite resin restorations using different bonding agents and found that clear fill standard error (SE) bond has highest bond strength. A second consideration is the ability to isolate the cavity preparation. Therefore, in the present study, foundation materials were filled in both ideal and contaminated conditions, as contamination may affect the long-term prognosis of a restoration. However, in the present study, no microleakage was observed with either amalgam or nanocomposites both in ideal and contaminated conditions. In the present study, the CAD-CAM milled metal crowns were used to cement over the restored tooth samples. Previous studies have shown that CAD-CAM crowns exhibit superior dimensional stability and marginal adaptation than conventional crowns regardless of the luting cement used [13]. The milling technique is unique way to create or produce a shape with the help of special cutter or burs from a block of material [25]. In this study, metal crown milling was done with the help of five milling axes machines. The benefit of this is precise fitting of crowns over the samples and production of accurate shape and margins. Various previous studies have reported impressive results for metal crowns manufactured by milling technique. The use of a digital technique for fabrication of crowns has also helped in overcoming the inherent problems associated with convention fabrication of crown wax-up, mixing of investment, casting, etc. [26,27,28].

Studies have shown that cements used for cementation have a significant impact on the microleakage and caries development. Luting cements having desirable physical and mechanical properties is another factor determining the success of extra coronal restoration. The clinician can select from many of the luting cements currently used in dentistry depending upon the situation of the patient. In the present study, resin-reinforced GIC and self-adhesive resin cements were used for luting both in ideal and contaminated conditions. This study emphasized that maintaining a dry field diminishes the microleakage. This study also demonstrated that the resin cement is better than resin reinforced GIC under both ideal and contaminated conditions with all crown foundation material groups. In the present study, we followed proper standardization of the test specimens and techniques. Single operator done the tooth preparation and following procedures to reduce the inter operator variations. The metal crowns were prepared by CAD-CAM procedure so as to avoid errors and variation due to manual techniques involved. The aging process was performed by thermocycling unit uniformly. However, still there are certain limitations of the present study, first and foremost it is an in vitro study. The results of the study should be should be verified in clinical scenario. Use of SEM or more sophisticated techniques such as confocal microscopy may be recommended for linear measurements using photosensitive pigmenting agents. Comparison with ceramic crowns is also recommended in future studies.

## 5. Conclusions

Within the limitations of the present in vitro study it can be concluded that microleakage can be prevented under restored teeth by using proper crown foundation material with self-adhesive resin cements. In the present study, composites and amalgam both showed less microleakage, when used as crown foundation material with self-adhesive resin cement (in both ideal-RGr-1,3 or contaminated-RGr-2,4) than in resin reinforced glass ionomer cement in tooth samples luted with CAD-CAM metal crown. Overall, the self-adhesive resin cement showed comparatively reduced microleakage in all combination with different crown foundations. There was no microleakage under the crown foundation either with amalgam/cavity varnish or nanocomposite/dentinal bonding agent combination both in ideal or contaminated condition. Microleakage was observed when extra coronal restorations were cemented without foundation (RG-5). Thus, this combination can be used in daily clinical practice to provide better protection from further decay.

## Figures and Tables

**Table 1:** Comparison of microleakage between different crown foundation materials on facial surface (n = 20).

Foundation		Number (n)	Mean (μm)	Standard Deviation (μm)	Critical Value	*p*-Value
RGr-1	Amalgam/cavity varnish, ideal	20	0.00	0.00	22.024	<0.001 *
RGr-2	Amalgam/cavity varnish, contaminated	20	0.00	0.00		
RGr-3	Nanocomposite/dentinal bonding agent, ideal	20	0.00	0.00		
RGr-4	Nanocomposite/dentinal bonding agent, contaminated	20	0.00	0.00		
RGr-5	No foundation (Control)	20	12.52	22.44		

* *p*-value < 0.001 significant.

**Table 2:** Comparison of microleakage between different luting cements on experimental side of facial surface (n = 25).

		Facial Surface (Experimental Side)				
		Number(n)	Mean (μm)	Standard Deviation (μm)	Critical Value	*p*-Value
CGr-1	Resin Reinforced GIC—Ideal	25	137.64	33.71	13.980	<0.001 *
CGr-2	Resin Reinforced GIC—contaminated	25	211.01	80.95		
CGr-3	Self-adhesive Resin Cement, Ideal	25	119.78	36.82		
CGr-4	Self-adhesive Resin Cement, Contaminated	25	150.42	46.47		

* *p*-value < 0.001 significant.

## Data Availability

Data can be made available on demand by chief researcher for academic purpose by email.

## References

[1] Venes D. (1993). Taber’s Cyclopedic Medical Dictionary.

[2] Going R.E., Sawinski V.J. (1966). Microleakage of a new restorative material. J. Am. Dent. Assoc..

[3] Cox C.F., Keall C.L., Keall H.J., Ostro E., Bergenholtz G. (1987). Biocompatibility of surface-sealed dental materials against exposed pulps. J. Prosthet. Dent..

[4] Herrero A.A., Yaman P., Dennison J.B. (2005). Polymerization shrinkage and depth of cure of packable composites. Quintessence Int..

[5] Hilton T.J., Schwartz R.S., Ferracane J.L. (1997). Microleakage of four class II resin composite insertion techniques at intraoral temperature. Quintessence Int..

[6] Anusavice K.J., Shen C., Rawls H.R. (2003). Phillips’ Science of Dental Materials.

[7] Beun S. (2007). Characterization of nanofilled compared to universal and microfilled composites. Dent. Mater..

[8] Tjan A.H., Chiu J. (1989). Microleakage of core materials for complete cast gold crowns. J. Prosthet. Dent..

[9] Platt J.A. (1999). Resin cements: Into the 21st century. Compend. Contin. Educ. Dent..

[10] Anusavice K.J., Hojjatie B. (1992). Tensile stress in glass-ceramic crowns: Effect of flaws and cement voids. Int. J. Prosthodont..

[11] Vargas-Belón K., Chambilla-Torres K., Sánchez-Tito M. (2022). Comparison of marginal microleakage of metal copings cemented with three luting cements. J. Clin. Exp. Dent..

[12] Scholz K.J., Tabenski I.M., Vogl V., Cieplik F., Schmalz G., Buchalla W., Hiller K.A., Federlin M. (2021). Randomized clinical split-mouth study on the performance of CAD/CAM-partial ceramic crowns luted with a self-adhesive resin cement or a universal adhesive and a conventional resin cement after 39 months. J. Dent..

[13] Robaian A., Maawadh A., Alghomlas Z.I., Alqahtani A.M., Alothman T.A., Alhajri F.F., Albar N. (2021). Evaluation of the marginal microleakage of CAD-CAM compared with conventional interim crowns luted with different types of cement: An in-vitro study. Niger. J. Clin. Pract..

[14] Nelsen R.J., Wolcott R.B., Paffenbarger G.C. (1952). Fluid Exchange at the Margins of Dental Restorations. J. Amer. Dent. Assoc..

[15] Kidd E.A.M. (1976). Microleakage: A review. J. Dent..

[16] Eick J.D., Welch F.H. (1986). Polymerization shrinkage of posterior composite resins and its possible influence on postoperative sensitivity. Quintessence Int..

[17] Krejci I., Lutz F. (1991). Marginal adaptation of class V restorations using different restorative techniques. J. Dent..

[18] Mitra S.B., Dong W.U., Holmes B.N. (2003). An application of nanotechnology in advanced dental materials. J. Am. Dent. Assoc..

[19] Barber D., Lyell J., Massler M. (1964). Effectiveness of copal resin under amalgam restorations. J. Prosthet. Dent..

[20] Andrews J.T., Hembree J.H. (1978). Microleakage of several amalgam systems: An animal study. J. Prosthet. Dent..

[21] Ebadian B., Fathi A., Savoj M. (2021). In vitro evaluation of the effect of different luting cements and tooth preparation angle on the microleakage of zirconia crowns. Int. J. Dent..

[22] Bagis Y.H., Baltacioglu I.H., Kahyaogullari S. (2009). Comparing microleakage and the layering methods of silorane-based resin composite in wide class II MOD cavities. Oper. Dent..

[23] Eakle W.S., Ito R.K. (1990). Effect of insertion technique on microleakage in mesio-occlusodistal composite resin restorations. Quintessence Int..

[24] Ansari A.A. (2004). An evaluation of strength of composite resin restorations using different bonding agents—An in-vitro study. J. Indian Soc. Pedod. Prev. Dent..

[25] Bindl A., Mörmann W.H. (2003). Clinical and SEM evaluation of all-ceramic chair-side CAD/CAM-generated partial crowns. Eur. J. Oral Sci..

[26] Nakamura T., Dei N., Kojima T., Wakabayashi K. (2003). Marginal and internal fit of Cerec 3 CAD/CAM all-ceramic crowns. Int. J. Prosthodont..

[27] Chaturvedi S., Alqahtani N.M., Addas M.K., Alfarsi M.A. (2020). Marginal and internal fit of provisional crowns fabricated using 3D printing technology. Technol. Health Care.

[28] Quante K., Ludwig K., Kern M. (2008). Marginal and internal fit of metal-ceramic crowns fabricated with a new laser melting technology. Dent. Mater..

